# The Effects of Soy Protein and Cocoa With or Without Isoflavones on Glycemic Control in Type 2 Diabetes. A Double-Blind, Randomized, Placebo-Controlled Study

**DOI:** 10.3389/fendo.2019.00296

**Published:** 2019-05-09

**Authors:** Judit Konya, Thozhukat Sathyapalan, Eric S. Kilpatrick, Stephen L. Atkin

**Affiliations:** ^1^Diabetes Research Centre, University of Hull, Kingston upon Hull, United Kingdom; ^2^Sidra Research Centre, Doha, Qatar; ^3^Weill Cornell Medicine, Doha, Qatar

**Keywords:** type 2 diabetes, soy, cocoa, glycemia control, isoflavones

## Abstract

**Objective:** Soy and cocoa have been suggested to be beneficial for diabetes. The aim of this study was to identify the effects of soy protein, isoflavones, and cocoa on glycemic control parameters.

**Research design and methods:** The study was a parallel, double-blind, placebo-controlled study where patients with diet or metformin controlled type 2 diabetes were randomized to, casein soy protein with or without isoflavones (SPI, SP), and with or without cocoa (SPIC, SPC) arms for an 8 week period. Glycemic control and cardiovascular risk factors were assessed prior to and after the completion of the dietary intervention. Sixty participants completed the study.

**Results:** Soy protein improved HbA1c compared to casein (*p* < 0.05). The addition of isoflavones improved indices of insulin resistance and LDL [delta QUICKIE (SPI: −0.12 ± 0.04 vs. SP: 0.03 ± 0.06, *p* = 0.03); delta LDL (−0.27 ± 0.41 vs. 0.22 ± 0.43, *p* = 0.02); percentage change in HOMA (31.02 ± 54.75 vs. −14.42 ± 27.07, *p* = 0.02); percentage change in QUICKIE (−3.89 ± 7.07 vs. 6.11 ± 10.54, *p* = 0.01)]. However, the addition of cocoa provided no benefit with or without isoflavones.

**Summary:** Soy protein had intrinsic activity on glycemic control compared to casein. Isoflavones improved both insulin resistance and LDL, but cocoa did not have added benefit on these indices.

**Clinical Trial Registration:** www.ClinicalTrials.gov, identifier NCT01754662.

## Introduction

The number of patients with diabetes is projected to rise to 592 million in the next 25 years (1). The prevalence of cardiovascular disease is three times higher in patients with type 2 diabetes (2) with a four-to six-fold higher mortality (3).

Lifestyle and diet modification remains the first step in improving glycemic control and soy has been shown to improve insulin resistance in both primate studies and post-menopausal women (4–7). Other animal studies have suggested that diets containing soy result in an improvement in insulin sensitivity, glycemic control and a decrease in fasting insulin levels (8–11). *In vitro* data suggests that the glycemic action of soy phytoestrogens may be due to their α-glucosidase inhibitory effects, inhibition of the glucose uptake at the intestinal brush border, and a tyrosine kinase inhibitory action (12–14).

Consumption of soy has been suggested to have an inverse relationship with mortality from CVD perhaps through a favorable effect on lipid levels and glycemic control. However, a meta-analysis of various soy preparations with a wide range of soy protein and isoflavone intake, did not support LDL or HDL cholesterol changes in response to soy-associated isoflavones (15), and isoflavones given alone also appeared to be ineffective (16). However, a major confounding issue is that all of the studies to date have been undertaken with soy protein that will also contain isoflavones and there are no studies with soy protein alone that is confirmed to be free from isoflavones.

Cocoa, with high polyphenol content, is potentially beneficial for patients with type 2 diabetes with improvement of cholesterol levels, blood pressure, insulin resistance, and overall cardiovascular risk reduction (17–20). A meta-analysis concluded that chocolate or cocoa improve flow-mediated vasodilatation, fasting insulin, and insulin resistance (21). A recent study reported that an isoflavones and flavanols product over a 1-year period improved multiple cardiovascular risk factors in post-menopausal women (22), and others reported an improvement in endothelial dysfunction in diabetes patients given high polyphenol chocolate (23), though another study found only a change in HDL.

Given the limited data on the combination of soy protein isoflavones and cocoa on glycemic control and cardiovascular risk parameters in type 2 diabetes, this study was therefore undertaken.

## Research Design and Methods

### Patients

Eighty-four patients with diet- or metformin controlled type 2 diabetes aged 45–80 were consecutively recruited to the study through routine diabetes clinics and local media advertisement. Seventy patients were randomized and sixty patients completed the study (Supplementary Information).

The diagnosis of diabetes was made according to the WHO guidelines (24). Patients were either diet controlled (*n* = 24) or on stable metformin therapy (*n* = 36) for at least 3 months before study commencement, and medication was not altered over the study period.

Inclusion criteria were type 2 diabetes on diet alone or stable metformin therapy, men, and post-menopausal women. Exclusion criteria: premenopausal women, women on hormonal replacement therapy within the preceding 6 months, smokers, vegans, vegetarians, patients with regular soy consumption, and patients with allergy to any nutritional component of the study bars were excluded during the screening process. Antibiotic treatment within the previous 3 months, or during the study, was an exclusion criterion as antibiotics have been shown to alter isoflavone metabolism and absorption through interference with gut flora (25). Concomitant participation in any other interventional medical trial was not allowed.

All subjects gave written informed consent in accordance with the International Conference of Harmonization Good Clinical Practice (ICH GCP) and the Declaration of Helsinki (ClinicalTrials.gov Identifier: NCT01754662). The protocol was approved by the Humber Bridge Regional Ethics Committee (11/YH/0219). The conduct of the trial was in accordance with all relevant legislation.

### Study Protocol

This was a randomized, parallel, double-blind, placebo-controlled clinical trial. Following informed consent and eligibility screening, patients met with a dietitian who explained the need to avoid dietary products with a high isoflavone-content and to maintain their current diet and level of physical activity. Blood samples were taken after an overnight fast during Visit 2 (week 2) and Visit 4 (week 10). Weight and blood pressure were also recorded. Compliance was checked by collecting and counting empty wrappers, and uneaten bars.

The randomization was performed by Essential Nutrition Ltd, UK. A computer generated randomization list was used to provide balanced blocks of patient numbers for each of the groups. A 1:1:1:1:1 treatment allocation was used without revealing the block size. Patients were randomized consecutively during Visit 2 by the study staff, when the next available randomization number was assigned.

### Intervention

Patients were randomized to casein protein as placebo (P) (*n* = 11), soy protein alone that was isoflavone free (SP) (*n* = 15), soy protein + isoflavones (SPI) (*n* = 16), soy protein + cocoa (SPC) (*n* = 13), or soy protein+isoflavones + cocoa (SPIC) (*n* = 15) groups.

Two bars containing a base of 7.5 g (15 g daily) of 70% isolated soy protein powder (Solcon F; CHS Ashdod, Israel) with or without added isoflavones (Solgen 16 mg per bar, 32 mg in total daily; CHS Ashdod, Israel) with or without 400 mg of cocoa polyphenols in 1.6 g of cocoa powder (CocoanOX 12%, Natraceutical S. A., Barcelona, Spain) were given twice daily (mid-morning and mid-afternoon) for 8 weeks following a 2 week run-in period. The isolated soy powder had the isoflavones removed by repeat 95% alcohol extraction (Dishman Ltd, UK) to give an isoflavone content of <300 parts per billion (Assayed by FERA, Sand Hutton, UK). Casein was used as the comparator protein (Halo Foods Ltd., Swansea, UK). All the bars used in the study were matched for taste and macronutrient content (Halo Foods Ltd., Swansea, UK). Randomization and labeling of the trial supplies were done by Essential Nutrition, Brough, UK.

### Study Measurements

Fasting venous blood samples were collected into serum gel, EDTA, and fluoride oxalate Vacutainer tubes. Samples for insulin were spun down at 3,500 g for 15 min at 4°C, within 30 min after drawing the samples, then were stored at −80°C until analysis. Glucose, total cholesterol, triglyceride, HDL cholesterol levels were analyzed using an enzymatic method (Synchron LX20 analyzer, Beckman-Coulter, High Wycombe, UK). Friedewald equation was used to determine LDL cholesterol levels ([LDL-chol] = [Total chol]–[HDL–chol]–[TG]/2.2). Serum insulin was measured by competitive chemiluminescent immunoassay (DPC Immulite 2000 analyzer, Euro/DPC, Lanberis, UK) where coefficient of variation was 8% with an analytical sensitivity of 2 μU/ml without cross-reactivity with proinsulin. Insulin resistance (IR) was calculated using the Homeostasis Model Assessment (HOMA) calculation (HOMA-IR=[insulin×glucose]/22.5) (26). The quantitative insulin sensitivity check index (QUICKI) was calculated using the following formula: QUICKI = 1/(log[fasting insulin μU/mL] + log[fasting glucose mg/dL]) (27). HbA1c was measured using Menarini HA-8160 analysers (Menarini Diagnostics Limited).

### Statistical Analysis

The sample size calculation was based on the effect of soy phytoestrogen in post-menopausal women with diabetes (28) and was performed using N-Query Advisor 5.0 (Statistical Solutions, Cork, Ireland). Powered specifically for HOMA, the minimum difference worth detecting/observed difference was 1.0, estimated within group SD was 0.9; therefore, for 80% power and a significance level of 5%, a sample size of 10 per group was calculated.

Results are expressed as mean ± SD where applicable. Mean percentage changes obtained at the end of the treatment phase at 2 month were compared with the baseline results, using the one way ANOVA with Tukey' *post-hoc* testing.

Statistical analysis was performed using SPSS 22 (IBM Corp., New York, NY) and statistical significance was defined as (*p* < 0.05).

## Results

### Baseline Characteristics

Sixty of the seventy patients who were randomized completed the trial. Baseline patient characteristics were comparable between the groups (Table 1).

**Table 1:** Patient group characteristics.

	**SPI**	**SP**	**SPC**	**SPIC**	***P***	**Overall**
*N*	13	13	12	11	11	60
Gender (male, female)	11, 2	7, 6	10, 2	10, 1	9, 2	47, 13
Age (year)	63.46 ± 6.33	66.77 ± 8.35	64.42 ± 9.53	66.91 ± 10.28	61.82 ± 7.93	64.7 ± 8.46
Diabetes control (diet, metformin)	4, 9	4, 9	5, 7	7, 4	4, 7	24, 36
Lipid treatment (statin, no statin)	8, 5	11, 2	10,2	5, 6	9, 2	43, 17
Diabetes duration (year)	4.96 ± 4.2	3.81 ± 3.16	4.98 ± 4.30	5.14 ± 4.48	3.99 ± 3.96	4.57 ± 3.93

*SPI, soy protein + isoflavones; SP, soy protein; SPC, soy protein + cocoa; SPIC, soy protein + isoflavones + cocoa; P, placebo*.

### Cardiovascular Markers, Lipids, and Glycemic Control

The casein protein was used as a comparator and control for the soy protein with the null hypothesis that there would be no difference between the two proteins and that this would allow these two groups to be combined as the placebo comparator for the study. However, soy protein alone had a significant effect on the percentage improvement in HbA1c (*p*=0.046) rejecting the null hypothesis. Therefore, to account for the additive affect of soy protein in the analysis, the casein arm was excluded from the one-way ANOVA analysis and comparison of the soy protein alone with the other soy containing arms of the study was performed.

One-way ANOVA between the groups showed statistical difference in percentage change in weight [*F*_(3, 56)_ = 3.12, *p* = 0.03], percentage change in BMI [*F*_(3, 56)_ = 3.61, *p* = 0.02], percentage change in LDL [*F*_(3, 45)_ = 2,94, *p* = 0.04] and in percentage change in QUICKI [*F*_(3, 53)_ = 2.86, *p* < 0.05]. A Tukey *post-hoc* test revealed that weight and BMI decreased significantly in the SP group compared to the SPC group (both *p* = 0.05), while LDL and QUICKI improved significantly in the SPI group compared to the SP group (*p* = 0.03 and *p* < 0.05).

In comparison with SPI there was a significant change in the SP group with an improvement in delta QUICKIE (SPI: −0.12 ± 0.04 vs. SP: 0.03 ± 0.06, *p* = 0.03), delta LDL (−0.27 ± 0.41 vs. 0.22 ± 0.43, *p* = 0.02), percentage change in HOMA (31.02 ± 54.75 vs. −14.42 ± 27.07, *p* = 0.02), and percentage change in QUICKIE (−3.89 ± 7.07 vs. 6.11 ± 10.54, *p* = 0.01) (Table 2).

**Table 2:** Study results.

	**SPI**			**SP**			**SPC**			**SPIC**		
	**0 months**	**2 months**	**% change**	**0 months**	**2 months**	**% change**	**0 months**	**2 months**	**% change**	**0 months**	**2 months**	**% change**
Weight (kg)	93.56 ± 19.71	93.38 ± 19.90	−0.24 ± 2.09	89.26 ± 12.86	88.60 ± 12.74	−0.73 ± 1.39	100.33 ± 26.99	100.54 ± 26.28	0.40 ± 1.40	99.07 ± 20.64	99.62 ± 19.93	0.73 ± 2.00
BMI (kg/m2)	31.00 ± 5.63	30.96 ± 5.87	−0.23 ± 2.28	30.91 ± 4.54	30.64 ± 4.65	−0.95 ± 1.62	32.76 ± 7.61	32.83 ± 7.34	0.40 ± 1.40	31.69 ± 5.27	31.94 ± 5.0	0.97 ± 2.27
Systolic BP(Hgmm)	133.62 ± 21.38	134.08 ± 17.92	0.95 ± 6.85	141.3 ± 27.88	137.08 ± 26.73	−2.01 ± 13.88	138.42 ± 12.67	142.67 ± 20.69	3.27 ± 13.83	137.36 ± 18.47	143.27 ± 18.57	5.19 ± 14.45
Diastolic BP (Hgmm)	77.54 ± 10.14	81.69 ± 10.50	5.95 ± 11.77	76.77 ± 4.11	76.31 ± 15.57	−0.71 ± 18.59	78.08 ± 6.60	80.67 ± 12.26	3.26 ± 13.30	77.55 ± 14.28	80.27 ± 9.06	6.53 ± 23.43
Cholesterol (mmol/l)	4.31 ± 1.01	4.11 ± 0.88	−4.53 ± 7.06	3.85 ± 0.53	3.93 ± 0.51	2.64 ± 11.59	3.91 ± 1.10	3.93 ± 0.95	3.38 ± 11.87	4.68 ± 1.64	4.68 ± 1.54	0.30 ± 9.36
Triglicerides (mmol/l)	1.46 ± 0.62	1.46 ± 0.87	−0.19 ± 27.15	1.54 ± 0.79	1.37 ± 0.51	−4.03 ± 23.42	1.47 ± 0.9	1.42 ± 0.66	5.25 ± 27.51	1.84 ± 1.65	1.88 ± 1.72	5.96 ± 26.42
HDL (mmol/l)	1.24 ± 0.31	1.18 ± 0.31	−2.5 ± 6.76	1.2 ± 0.34	1.21 ± 0.35	−1.03 ± 9.76	1.16 ± 0.23	1.13 ± 0.22	1.36 ± 7.56	1.13 ± 0.28	1.10 ± 0.27	−3.07 ± 8.98
LDL (mmol/l)	2.38 ± 1.19	2.23 ± 1.00	−11.36 ± 15.58	1.93 ± 0.56	2.14 ± 0.55	14.42 ± 24.98	2.17 ± 0.96	2.18 ± 0.98	0.18 ± 20.46	2.54 ± 0.88	2.59 ± 0.90	3.56 ± 17.22
Chol/HDL ratio	3.74 ± 1.05	3.62 ± 0.89	−2.59 ± 7.70	3.41 ± 0.90	3.58 ± 1.01	5.20 ± 15.46	3.61 ± 1.00	3.59 ± 0.99	0.21 ± 13.11	4.16 ± 1.32	4.22 ± 1.09	3.83 ± 15.16
HbA1c (%)	6.42 ± 0.53	6.23 ± 0.58	−1.95 ± 2.85	6.22 ± 0.57	6.17 ± 0.48	−0.67 ± 4.15	6.31 ± 0.59	6.24 ± 0.60	−1.14 ± 2.23	6.91 ± 0.87	6.62 ± 1.03	−4.03 ± 8.74
HbA1c (mmol/mol)	46.77 ± 5.90	45.38 ± 6.21	−3.00 ± 4.16	44.69 ± 6.17	44.08 ± 5.25	−0.97 ± 5.98	45.36 ± 6.33	44.64 ± 6.44	−1.60 ± 3.10	51.5 ± 9.80	48.88 ± 11.28	−4.67 ± 12.75
Fasting glucose (mmol/l)	6.69 ± 1.18	7.2 ± 1.78	7.03 ± 12.27	6.39 ± 1.15	6.27 ± 1.20	−1.68 ± 10.43	6.56 ± 1.32	6.54 ± 1.36	−0.19 ± 7.41	6.65 ± 1.34	7.37 ± 2.51	10.11 ± 25.79
Fasting insulin (mIU/l)	19.07 ± 12.55	22.70 ± 15.19	20.16 ± 39.74	19.20 ± 9.42	15.75 ± 7.30	−16.01 ± 24.92	13.06 ± 6.57	14.35 ± 7.48	11.69 ± 25.81	20.72 ± 9.85	39.34 ± 64.63	56.50 ± 170.55
HOMA-IR	5.53 ± 3.22	7.52 ± 5.84	31.02 ± 54.75	5.41 ± 3.14	4.54 ± 2.33	−14.42 ± 27.07	3.72 ± 1.74	4.11 ± 2.10	11.90 ± 30.22	6.44 ± 3.70	17.22 ± 33.20	112.47 ± 329.79
QUICKI	0.50 ± 0.07	0.48 ± 0.08	−3.89 ± 7.07	0.50 ± 0.05	0.53 ± 0.10	6.11 ± 10.54	0.54 ± 0.07	0.53 ± 0.07	−1.33 ± 6.54	0.49 ± 0.09	0.48 ± 0.10	−2.96 ± 13.25

*One-way ANOVA between the groups showed statistical difference in percentage change in weight [F_(3, 56)_ = 3.12, p = 0.03], percentage change in BMI [F_(3, 56)_ = 3.61, p = 0.02], percentage change in LDL [F_(3, 45)_ = 2,94, p = 0.04] and in percentage change in QUICKI [F_(3, 53)_ = 2.86, p < 0.05]. A Tukey post-hoc test revealed that weight and BMI decreased significantly in the SP group compared to the SPC group (both p = 0.05), while LDL and QUICKI improved significantly in the SPI group compared to the SP group (p = 0.03 and p < 0.05). Results are presented as mean ± SD. BMI, body mass index; BP, blood pressure; HDL, high density lipoprotein; LDL, low density lipoprotein; Chol/HDL ratio, cholesterol/HDL ratio; HbA1c, Hemoglobin A1c; HOMA-IR, homeostatic model assessment of insulin resistance; QUICKI, quantitative insulin sensitivity check index*.

## Discussion

In this short term study there were significant changes in both LDL concentration and in insulin resistance (QUICKI) in the patient group who consumed soy protein with isoflavones, compared to isoflavone-free soy protein. Patients who consumed soy protein only lost weight compared to those who consumed cocoa enriched soy protein bars.

We have previously shown that 132 mg of isoflavones alone for 3 months in a crossover study in post-menopausal women with type 2 diabetic had no effect on glycemic control or any cardiovascular parameter measured (16). However, when that same dose of isoflavone was given in combination with 30 g of soy resulted in significant improvement in HbA1c, fasting insulin, HOMA-IR, cholesterol, and LDL levels (28). In the present study we found that SP without isoflavones in comparison to casein, improved glycemic control with a change in HbA1c, the mechanism of which remains unclear. However, in a study comparing whey protein and soy isolate effects on an oral glucose tolerance test, soy isolate resulted in decreased peak blood glucose and higher insulin responses that may then reflect in improved post-prandial glycemic control and an improved HbA1c (29).

In a randomized parallel study with obese patients with type 2 diabetes, it was shown that a soy-based diet improved weight and glycemic control more significantly than a control diet (30). In our short term study, weight changed minimally, though significantly in the SP group when compared to the SPC group.

This study was of short duration to focus on glycemic control changes and therefore not designed to look specifically at cardiovascular risk indices. However, this study showed improvement in LDL in the SPI group when compared to the SP group, in accord with a meta-analysis that suggested that the magnitude of the favorable change in serum cholesterol and LDL levels may be affected by the baseline degree of hypercholesterolemia (31). Whilst markers of inflammation were not measured in this study, it has been noted that inflammatory markers such as CRP are reduced by soy with isoflavones in type 2 diabetes, and therefore an effect on inflammation may have an indirect and positive effect on glycemic control (32).

A recent study showed improvement in insulin resistance measured by HOMA-IR and QUICKI, fasting glucose and insulin, fasting triglycerides, LDL cholesterol, HDL:LDL, and total cholesterol:HDL ratios in post-menopausal women who consumed a food product with isoflavones and flavanols over a 1-year period, compared to placebo (33). In our shorter duration study period, we found that any beneficial changes may be lost by adding cocoa to the study preparations, suggesting that the positive findings reported by others may have been abrogated by the flavanol preparation (22).

The main limitation of the study was the relatively short duration and the low number of patients recruited. The other limitation is that the cocoa and soy preparations vary between the different research studies, therefore the comparison of the results to the literature is limited due to the difference in the quality of the dietary products used by others.

In summary, soy protein without isoflavones had intrinsic activity on glycemic control compared to casein with a reduction in HbA1c. Soy alone was associated with a decrease in weight and BMI, and soy with isoflavones improved both insulin resistance and LDL; however, the addition of cocoa did not add further benefits for glycemic control, insulin resistance, or the lipid parameters.

## Author's Note

This work was part of an MD thesis and its publishing is in line with the University of Hull policy, and can be accessed online (34).

## Author Contributions

SA, TS, and EK devised the study and provided support with the planning, conduct of the study, and data analysis. JK recruited the patients, conducted study visits, collected, and analyzed the data. All authors contributed to the writing and revision of the manuscript.

### Conflict of Interest Statement

The authors declare that the research was conducted in the absence of any commercial or financial relationships that could be construed as a potential conflict of interest.
