# Supplementary figures and images for: The Effects of Soy Protein and Cocoa With or Without Isoflavones on Glycemic Control in Type 2 Diabetes. A Double-Blind, Randomized, Placebo-Controlled Study
Source: Front Endocrinol (Lausanne). 2019 May 9;10:296. doi: 10.3389/fendo.2019.00296 (PMC6521701; doi:10.3389/fendo.2019.00296)

Supplementary information

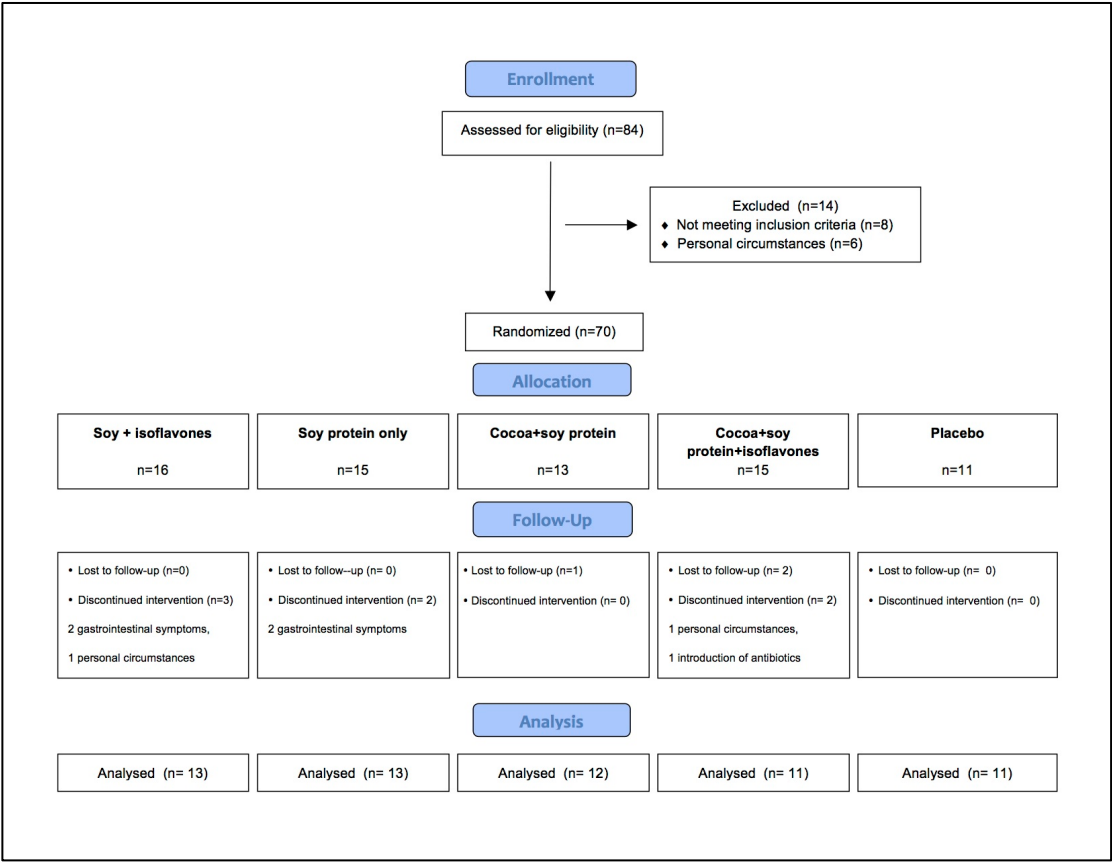

Recruitment and patient completion during the study period.

Supplement: Supplementary file 1 [file Data_Sheet_1.PDF]
